# Diagnostic and Monitoring Value of 2D Shear Wave Elastography in Subacute Thyroiditis: A Prospective Case–Control Study

**DOI:** 10.1155/ije/4035441

**Published:** 2026-09-19

**Authors:** Ioana Golu, Melania Balas, Mervat Matei, Toma Andreea-Daniela, Ana Corlan, Daniel Luches, Dana Amzar, Natalia Milos, Ioan Sporea, Alina Popescu, Mihaela Vlad

**Affiliations:** ^1^ 2nd Department of Internal Medicine – Discipline of Endocrinology, “Victor Babes” University of Medicine and Pharmacy, Timisoara, Romania, umft.ro; ^2^ Department of Endocrinology, County Emergency Hospital, Timisoara, Romania, scju-cluj.ro; ^3^ Centre for Molecular Research in Nephrology and Vascular Disease, “Victor Babes” University of Medicine and Pharmacy, Timisoara, Romania, umft.ro; ^4^ Department of Sociology, Western University of Timisoara, Timisoara, Romania; ^5^ 2nd Department of Internal Medicine – Discipline of Gastroenterology, “Victor Babes” University of Medicine and Pharmacy, Timisoara, Romania, umft.ro; ^6^ Department of Gastroenterology, Emergency County Hospital, Timisoara, Romania, spitalmures.ro

**Keywords:** shear wave elastography, subacute thyroiditis, thyroid stiffness

## Abstract

**Purpose:**

This study aimed to analyze the applicability of different shear wave elastography (SWE) methods in the diagnosis, differentiation, and monitoring of subacute thyroiditis (SAT).

**Methods:**

This prospective study included 26 patients diagnosed with SAT and 40 healthy controls. All participants underwent conventional B‐mode ultrasonography followed by two‐dimensional shear wave elastography (2D‐SWE), using a 4–15 MHz linear transducer (Aixplorer, Supersonic Imagine, France). Thyroid stiffness was assessed in kilopascals (kPa) and evaluated at diagnosis, 4 weeks, and 8 weeks. Statistical analysis included *t*‐tests, repeated‐measures ANOVA, receiver operating characteristic curve analysis, and Youden Index calculation to determine optimal diagnostic cut‐off values.

**Results:**

Baseline thyroid stiffness in SAT patients was significantly higher than in controls (127.25 ± 54.06 kPa vs. 19.5 ± 7.1 kPa; *p* < 0.001). Stiffness values decreased over time, reaching 117 ± 44.67 kPa at 4 weeks and 57.17 ± 22.89 kPa at 8 weeks. An exploratory cutoff value of 37.2 kPa showed complete separation between groups within this specific cohort. Beyond baseline group differences, the most relevant finding was the longitudinal change in thyroid stiffness during clinical recovery, supporting the role of SWE as a quantitative imaging biomarker of inflammatory activity.

**Conclusions:**

2D‐SWE showed promising performance as a quantitative imaging biomarker for evaluating and monitoring SAT in this exploratory cohort. It provides valuable quantitative data on tissue stiffness, complementing conventional ultrasound and aiding clinical decision‐making during follow‐up.

## 1. Introduction

Subacute thyroiditis (SAT) is an inflammatory condition of the thyroid gland, most commonly associated with viral infections. Many patients report an upper respiratory tract infection occurring 2–8 weeks prior to the onset of thyroid‐related symptoms. According to epidemiological studies, the general incidence of SAT is 4.9 cases per 100,000/year [1]. Thyroiditis causes damage to the thyroid follicular cells, which results in the absence of synthesis of new thyroid hormones and the release of large amounts of preexisting triiodothyronine (T3) and thyroxine (T4) hormones contained within the thyroid follicle into the bloodstream. The histopathological changes are different from those found in Hashimoto’s thyroiditis [2].

The affected follicles are predominantly infiltrated with mononuclear cells and show disruption of the epithelium. The follicular changes progress to form granulomas, and the accompanying interstitial fibrosis and inflammatory reaction are present in varying degrees. Therefore, the lesions are uneven in distribution and vary from one area to another depending on their stage of development.

SAT is generally diagnosed after evaluation of both clinical and laboratory data. This is due to the fact that specific ultrasonographic features suggestive of SAT have not been clearly defined [3]. The most frequently described ultrasonographic appearance is that of hypoechoic areas, located unilaterally or bilaterally, without a round‐oval shape, with a vague outline, and with reduced vascularity and Doppler signal, while the volume of the thyroid gland can be increased or remains normal. Sometimes, the ultrasound appearance can lead to diagnostic confusion [4].

The differential diagnosis of SAT is mainly made with other forms of thyroiditis. Thyroid cancer and lymphoma are other differential diagnoses to consider in the less specific presentations of SAT [5].

Ultrasound elastography is a noninvasive imaging technology sensitive to mechanical characteristics of tissues—their elasticity and stiffness [6]. Various techniques have been developed, based on sequences of two‐dimensional images obtained in real time, after the application of a dynamic or slowly varying force (considered “quasistatic”). The principle of elastography is based on the measurement of tissue displacement by means of ultrasound. The degree of distortion of the ultrasound beam, determined by an external force (applied manually or automatically by the ultrasound probe), is quantified. All methods use signal processing to create an elasticity measurement or image [7].

Shear wave elastography (SWE) is a newer technique that uses shear wave propagation tracking to map the elastic characteristics of tissues.

The linear transducer is operated by the ultrasound technician without the need for additional pressure. This technique was first described in 2004 by Bercoff et al. [8]. Waves are generated in the imaging plane, and the speed of the waves increases with tissue stiffness. A color‐coded image is displayed together with the B‐mode image and alongside quantitative information on tissue elasticity in terms of shear wave velocity (expressed in m/s) or estimated tissue stiffness (expressed in kilopascals [kPa]).

Thyroid elastography has been intensively studied over the past decade to evaluate thyroid pathology [9–11], especially nodular goiters, with the goal of predicting malignancy [12–15]. There are limited data available for patients with SAT.

The aim of this study was to compare thyroid SWE measurements in healthy subjects and patients with SAT, to detect differences between them, and to answer the question of whether this method could straight away detect normal or abnormal thyroid tissue.

## 2. Material and Method

### 2.1. Subjects

This prospective case–control study included a final cohort of 66 participants: 26 patients with SAT (study group) and 40 healthy volunteers (control group). Initially, 38 consecutive patients fulfilling the inclusion criteria were prospectively enrolled. Following the baseline clinical, laboratory, ultrasonographic, and SWE evaluation, 12 patients did not return for the scheduled follow‐up visits and were therefore lost to follow‐up. Consequently, the final longitudinal analysis included the 26 patients with complete assessments at baseline, 4 weeks, and 8 weeks. Since this investigation was designed as a prospective exploratory study, all consecutive eligible patients presenting during the study period were considered for inclusion rather than determining the sample size through a formal a priori power calculation. The diagnosis of SAT was established based on a combination of clinical manifestations (neck pain and/or fever), elevated inflammatory markers (erythrocyte sedimentation rate [ESR] and C‐reactive protein [CRP]), suppressed thyroid‐stimulating hormone levels with elevated free thyroxine (FT4), and characteristic ultrasonographic findings.

Patients with SAT were recruited from the outpatient Endocrinology Clinic and the Endocrinology Department of our institution. Conventional B‐mode ultrasonography was performed immediately before SWE examination to assess thyroid volume and echotexture, identify characteristic inflammatory lesions, and exclude other thyroid disorders, including thyroid nodules and autoimmune thyroid disease.

All patients with SAT were treated according to a standardized institutional protocol consisting of oral prednisone 30 mg/day, followed by gradual tapering by 5 mg/week according to clinical response.

The control group comprised healthy volunteers recruited from hospital staff and medical students. Controls were frequency‐matched to the SAT group for age and sex (mean age 48.3 ± 11.3 years; female/male ratio 27/13). All control participants had no personal history of thyroid disease, normal thyroid function tests, and normal B‐mode thyroid ultrasonography, including normal echogenicity, absence of thyroid nodules, and no ultrasonographic features suggestive of autoimmune thyroid disease.

All procedures were conducted in accordance with the ethical standards of the institutional and national research committees and with the Declaration of Helsinki (1975, revised in 2008). The study protocol was approved by the local Ethics Committee (Approval No. 470/20.06.2024), and written informed consent was obtained from all participants before enrollment.

### 2.2. Ultrasound and SWE

B‐mode ultrasonography and SWE examinations were performed with the same machine, Aixplorer (Supersonic Imagine, France), using a 4–15 MHz high‐resolution linear transducer. B‐mode ultrasound was used to assess thyroid volume and echotexture and to confirm eligibility (normal thyroid in controls; inflamed areas in SAT). SWE was then performed during the same session. Examination was facilitated by the application of a proper amount of coupling gel. During transducer maneuvering, examiners applied minimal neck pressure to reduce compression of structures and the risk of image artifacts that could influence the results of SWE. The transducer was kept stationary during SWE acquisitions. The SWE image was displayed alongside the B‐mode image. After each thyroid lobe was captured in a longitudinal section, using the Q‐BOX over the obtained image, a color map was displayed, ranging from blue to red. Dark blue corresponds to soft thyroid tissue, while red indicates stiff thyroid tissue. A frozen image was then obtained with as few artifacts as possible.

A circular region of interest (ROI) was placed inside the elastogram, and the diameter of the circle was successively enlarged between 6 and 22 mm; however, the typical ROI used was approximately 10 mm. In patients with SAT, the ROI was systematically repositioned across the entire inflammatory lesion to identify the most representative area demonstrating consistently high stiffness on the elastography color map. Final measurements were obtained within this region while carefully avoiding the thyroid capsule, major intrathyroidal vessels, cystic or necrotic areas, calcifications, and obvious elastographic artifacts.

The default setting for the thyroid SWE scale (range 0 to ≥ 100 kPa) was used. Quantitative tissue stiffness data were automatically displayed on the screen. The parameters for tissue elasticity, expressed in kPa, provided by the system were the mean elasticity index (SWE‐Mean), maximum elasticity index (SWE‐Max), minimum elasticity index (SWE‐Min), and standard deviation of elasticity index (SWE‐SD), along with the diameter of the circle.

For each subject, three SWE measurements were obtained per thyroid lobe, and the mean values were calculated for analysis. The recorded parameters included SWE‐Mean, SWE‐Max, and SWE‐SD, while SWE‐Min was excluded due to inconsistent reliability. Patients with SAT underwent B‐mode ultrasonography and SWE at diagnosis, as well as at 4‐week and 8‐week follow‐up intervals. The collected data were compared and statistically analyzed.

All ultrasound examinations were performed by two endocrinologists with over 15 years of experience in thyroid ultrasonography, who were blinded to the patients’ clinical status. The examiners were blinded to each other’s measurements.

### 2.3. Statistical Analysis

The results were collected in a Microsoft Excel file. Statistical analysis was performed using SPSS (version 26) because of its ability to handle statistical procedures that we intended to implement (ANOVA, receiver operating characteristic [ROC] curves). Continuous variables were expressed as mean ± standard deviation (SD) for data with a normal distribution, providing a clear measure of central tendency and dispersion. For variables that did not follow a normal distribution, the data were presented as median with interquartile range to more accurately reflect the distribution characteristics.

The primary elasticity parameter used for diagnostic and longitudinal analysis was SWE‐Mean.

To compare the mean elasticity index (EI) values between the two independent groups—patients with SAT and healthy controls—a two‐tailed independent samples *t*‐test was applied. This test was deemed appropriate, as the EI values represent continuous data and the assumption of normal distribution was met. Normality was assessed using the Shapiro–Wilk test and confirmed by visual inspection of Q–Q plots. The results demonstrated a statistically significant difference between the groups, with the SAT group showing markedly higher EI values (mean = 127.25 kPa, SD = 54.06; *p* < 0.001).

To evaluate changes in EI over time within the SAT group, a one‐way repeated‐measures ANOVA was conducted, with measurements taken at three time points: baseline (diagnosis), 4 weeks, and 8 weeks post‐diagnosis. The assumptions for repeated‐measures ANOVA were verified: normality was again assessed using the Shapiro–Wilk test, and Mauchly’s test of sphericity was used to evaluate the sphericity assumption. When sphericity was violated, the Greenhouse–Geisser correction was applied. This analysis allowed us to determine whether treatment and disease progression over time were associated with significant changes in thyroid stiffness, as measured by EI.

Interobserver reproducibility was assessed using the intraclass correlation coefficient (ICC) based on paired SWE‐Mean measurements obtained independently by two experienced endocrinologists in the 26 patients with SAT. Examiners were blinded to each other’s measurements. A two‐way random‐effects model with absolute agreement (ICC(2,1)) was applied. Confidence intervals were estimated using bootstrap resampling (2000 iterations) implemented in Python.

ROC analysis was performed using MedCalc software (MedCalc Software Ltd., Ostend, Belgium) to evaluate the diagnostic performance of SWE‐Mean. The area under the curve (AUC) and corresponding 95% confidence intervals were estimated using bootstrap resampling (2000 iterations) with bias‐corrected and accelerated (BCa) intervals.

## 3. Results

A total of 66 subjects were evaluated by two‐dimensional shear wave elastography (2D‐SWE), out of which 26 patients were with SAT (study group) and 40 were healthy subjects (control group). The control group (*n* = 40) had a mean age of 48.3 ± 11.3 years with a female‐to‐male ratio of 27/13, which was comparable to the SAT group (45.55 ± 16.27 years; 22/4), with no statistically significant differences in age or sex distribution (*p* > 0.05).

The clinical and biological characteristics of the study group are shown in Table 1.

**TABLE 1:** Baseline characteristics of the studied cases.

Characteristics	
Mean age	45.55 ± 16.27
F/M ratio	22/4
Pain (number of cases)	26/26
Fever (number of cases)	18/26
Asthenia (number of cases)	22/26
Sweating (number of cases)	21/26
Weight loss (number of cases)	22/26
Goiter (number of cases)	24/26
ESR = 0–20 mm/h	78.33
Fibrinogen = 220–496 mg/dL	692.16
C‐reactive protein = < 0.5 mg/dL	18.82
TSH (0.43–4.68 µUI/mL)	0.007 ± 0.004
FT4 (12–22 pmol/L)	47.84 ± 19.50

For each subject, three measurements were performed, and the mean values for each of the elastography parameters provided by the device (SWE‐Mean, SWE‐Max, and SWE‐SD) were calculated. The results are shown in Table 2.

**TABLE 2:** Mean values of thyroid tissue EI obtained by SWE in the two groups of subjects.

Samples/variables	SWE means (kPa)	SWE max. (kPa)	SWE SD (kPa)
Control group			
Right thyroid lobe (RTL)	19.6 ± 6.6	33.4 ± 9.5	4.1 ± 1.4
Left thyroid lobe (LTL)	19.5 ± 6.8	33.2 ± 11.2	4.4 ± 1.7
*p* (RTL vs. LTL)	0.92	0.91	0.29
Average of both lobes (RTL–LTL)	19.5 ± 7.1	33.4 ± 9.5	4.3 ± 1.5
Study group (SAT)	127.25 ± 54.06	177.38 ± 56.09	50.52 ± 10.89
*p* (Control vs. SAT)	< 0.001	< 0.001	< 0.001

*Note:* mean: mean value; max: maximum value.

Abbreviations: LTL, left thyroid lobe; RTL, right thyroid lobe; SD, standard deviation; SWE, shear wave elastography.

^a^Values expressed as mean ± standard deviation.

Inflammatory markers were assessed at diagnosis and during follow‐up. Mean values of ESR, fibrinogen, and CRP are presented in Table 3. All patients received corticosteroid therapy (prednisone 30 mg/day) with gradual dose tapering over 4–6 weeks according to clinical response. A progressive decline in inflammatory markers was observed during follow‐up.

**TABLE 3:** Evolution of inflammatory markers and thyroid elasticity index (EI) over time in patients with subacute thyroiditis.

Parameter (normal range)	At diagnosis	After 4 weeks	After 8 weeks
ESR (mm/h) (0–20)	78.33	15.33	9.88
Fibrinogen (mg/dL) (220–496)	692.16	381.16	201.06
C‐reactive protein (mg/dL) (< 0.5)	18.82	7.36	1.18
Elasticity index—EI mean (kPa)	127.25 ± 54.06	117 ± 44.67	57.17 ± 22.89

In our study, the mean EI measured using elastography was 127.25 ± 54.06 kPa at the time of diagnosis, 117.00 ± 44.67 kPa at the 4‐week follow‐up, and 57.17 ± 22.89 kPa at the 8‐week follow‐up. These values indicate a progressive reduction in tissue stiffness over the treatment period. No statistically significant difference was observed between baseline and 4‐week measurements (*p* = 0.46).

A comparison between the patient group diagnosed with SAT and the healthy control group (mean EI: 19.5 ± 7.1 kPa) revealed significantly higher EI values in the affected patients (Student’s *t*‐test: *t* = 10.109, df = 25.42, *p* < 0.001), confirming the diagnostic relevance of elastography in this context.

Further pairwise comparisons using the Dunnett T3 post hoc test demonstrated statistically significant differences between the mean EI values:

• Between 4 weeks and 8 weeks post‐treatment initiation (Dunnett *T*3 = 6.11, *p* < 0.001, effect size *r* = 0.41),
• And between the baseline (diagnosis) and the 8‐week follow‐up (Dunnett *T*3 = 6.09, *p* < 0.001, *r* = 0.41).

**FIGURE 1:**
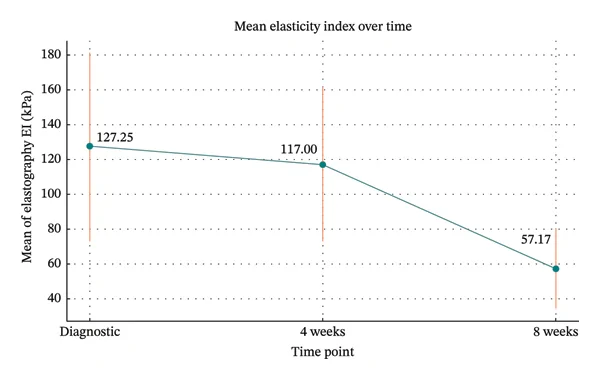
Mean elasticity index (EI) values (kPa) measured by elastography at baseline (diagnosis), 4 weeks, and 8 weeks after treatment initiation in patients with subacute thyroiditis.

These findings suggest a consistent and clinically meaningful decline in tissue stiffness as a response to treatment over time. These progressive changes in tissue elasticity, along with their statistical significance, are visually represented in Figure 1, illustrating the downward trend in mean EI values over the course of treatment.

Following the identification of significant differences in mean EI values across time points (Figure 1), we aimed to determine the optimal diagnostic cutoff value capable of distinguishing patients with SAT from healthy controls.

To achieve this, we generated a range of potential thresholds based on EI values and calculated the Youden Index for each. The Youden Index is defined as:

(1) Youden Index=Sensitivity+Specificity−1.

This metric identifies the threshold that maximizes the difference between the true positive rate and the false positive rate, thereby selecting the cutoff that offers the most favorable balance between sensitivity and specificity.

The analysis revealed that an EI threshold of 37.2 kPa yielded the maximum Youden Index value of 1.0, corresponding to perfect diagnostic performance—100% sensitivity and 100% specificity—within our dataset. This indicates a complete separation between patients with SAT and healthy controls based on the measured EI values.

**FIGURE 2:**
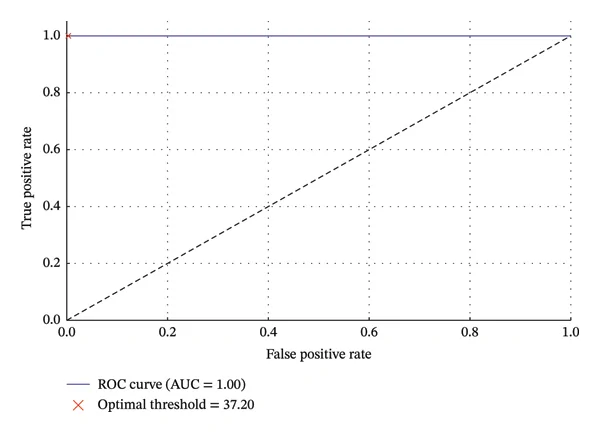
ROC curve illustrating the diagnostic performance of mean elasticity index (EI) values obtained via 2D‐SWE in distinguishing patients with subacute thyroiditis from healthy controls.

To visually represent this diagnostic performance, a ROC curve was constructed (Figure 2). The resulting AUC was 1.00, further confirming the excellent discriminative capacity of 2D‐SWE in this clinical context. The optimal threshold of 37.2 kPa is marked on the ROC curve, highlighting the point of maximal diagnostic efficiency.

These findings demonstrate strong discrimination between SAT patients and healthy controls within the present cohort; however, the proposed cutoff remains exploratory and requires external validation before clinical application.

**FIGURE 3:**
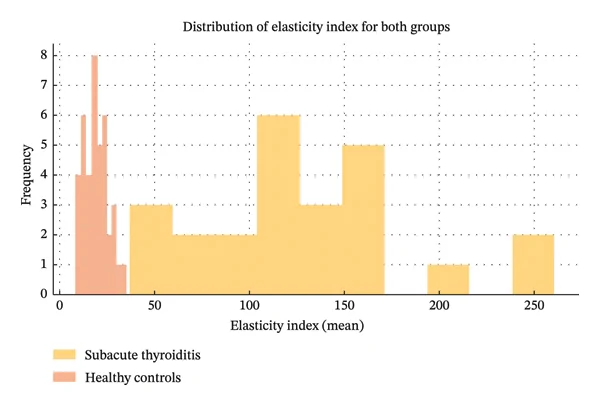
Histogram showing the distribution of elasticity index (EI) values for patients with subacute thyroiditis (yellow) and healthy controls (coral). The two groups demonstrate clearly separated, nonoverlapping distributions, with subacute thyroiditis patients exhibiting significantly higher EI values. This visual evidence supports the ROC curve findings (Figure 2) and explains the perfect diagnostic performance (AUC = 1.00) observed at the optimal threshold of 37.2 kPa.

The ROC analysis (Figure 2), yielding an AUC of 1.00 in this dataset, was further examined by analyzing the distribution of EI values across the two study groups. To illustrate this distribution, a histogram comparing EI values between patients with SAT and healthy controls was constructed (Figure 3).

This histogram reveals a clear bimodal distribution, with minimal to no overlap between the two populations. The healthy control group displays consistently low EI values, while the SAT group exhibits markedly elevated readings. The distinct separation between these distributions provides a visual and statistical justification for the high diagnostic accuracy observed in the ROC analysis and supports the robustness of the 37.2 kPa threshold determined via the Youden Index.

Taken together, Figures 2 and 3 not only reinforce the discriminative power of EI as a diagnostic biomarker but also highlight the reliability of SWE in detecting pathological changes in thyroid tissue. The absence of distributional overlap underlines the method’s potential for clinical application in early and accurate identification of SAT.

Interobserver agreement for SWE‐Mean was excellent (ICC = 0.997; 95% CI: 0.992–0.999), indicating near‐perfect reproducibility between the two examiners.

Measurement variability was further supported by a low coefficient of variation (CV = 2.65%). ROC analysis of SWE‐Mean demonstrated perfect discrimination between SAT patients and healthy controls (AUC = 1.00; 95% BCa CI: 1.00–1.00).

## 4. Discussions

2D‐SWE has emerged as a promising quantitative imaging technique for the assessment of thyroid tissue stiffness. While its diagnostic utility has been extensively investigated in thyroid nodules and diffuse autoimmune thyroid diseases, relatively few studies have explored its application in SAT, largely reflecting the lower prevalence of this condition [14, 15]. Because SAT induces profound inflammatory changes within the thyroid parenchyma, 2D‐SWE has the potential to objectively quantify alterations in tissue stiffness and to complement conventional clinical and ultrasonographic evaluation. Nevertheless, the available evidence remains limited, and further prospective studies are required to better define its role in the diagnosis and longitudinal monitoring of SAT.

The present prospective study demonstrates that thyroid stiffness is substantially increased during the acute inflammatory phase of SAT and progressively decreases during recovery. Importantly, the reduction in tissue stiffness lagged behind the improvement in inflammatory biomarkers, as no statistically significant decrease in SWE‐Mean was observed between baseline and the 4‐week follow‐up (*p* = 0.46), despite the marked decline in ESR and CRP. These findings suggest that 2D‐SWE may provide complementary information regarding structural tissue recovery that is not fully captured by conventional biochemical markers.

The marked increase in thyroid stiffness observed during the acute phase of SAT is biologically plausible and reflects the underlying pathological changes. Histologically, SAT is characterized by patchy destruction of thyroid follicles, mononuclear inflammatory infiltration, granulomatous inflammation with multinucleated giant cells, interstitial edema, and variable degrees of fibrosis [2]. These structural alterations modify the mechanical properties of thyroid tissue, resulting in increased stiffness within the affected regions. As inflammation resolves following treatment, thyroid elasticity progressively returns toward normal values, paralleling the restoration of normal tissue architecture.

Our study evaluated the utility of 2D SWE in assessing thyroid stiffness in patients with SAT compared to healthy individuals and in monitoring changes during an 8‐week follow‐up period. We found that thyroid stiffness was significantly higher in patients with SAT at diagnosis and showed a progressive reduction during follow‐up, in parallel with improvement in inflammatory markers. These findings support the clinical value of SWE as a noninvasive biomarker in both the diagnosis and monitoring of SAT. Our baseline mean EI in healthy controls (19.5 ± 7.1 kPa) aligns closely with values reported by Magri et al. (20.8 kPa), though slightly higher than those found by Bhatia et al. and Sebag et al. (13.6 and 15.9 kPa, respectively). These discrepancies likely stem from differences in ROI size, SWE system calibration, and transducer settings across studies [12, 16, 17].

In our study, the differences in EI between the two thyroid lobes are not significant in healthy subjects, so the examination of a single lobe is sufficient to characterize the mechanical characteristics of tissues of the gland.

The elastography image of SAT was first described by Ruchala et al., an image that demonstrated markedly increased stiffness of the affected thyroid regions [18]. Later, Ruchala et al. studied comparatively different types of diffuse thyroid impairment, including SAT [19]. The mentioned study included 2 patients with acute thyroiditis, 18 with SAT, 18 with chronic autoimmune thyroiditis (CAT), and 40 healthy subjects. Patients diagnosed with SAT were assessed three times: at baseline, at 4‐week follow‐up, and at 10 weeks following diagnosis and initiation of treatment. There were significant differences between the three measurements: 214.26 ± 32.5 kPa at baseline, 45.92 ± 17.4 kPa at 4 weeks, and 21.65 ± 5.3 kPa at 10 weeks. Thyroid stiffness was significantly higher at baseline in SAT compared to CAT (36.15 ± 18.7 kPa) or healthy controls (16.18 ± 5.4 kPa). Under treatment, values were restored to near normal.

Liu et al. also studied thyroid stiffness in SAT [13]. Both studies concluded the usefulness of SWE in the diagnosis of SAT and its differentiation from autoimmune thyroid diseases.

**FIGURE 4:**
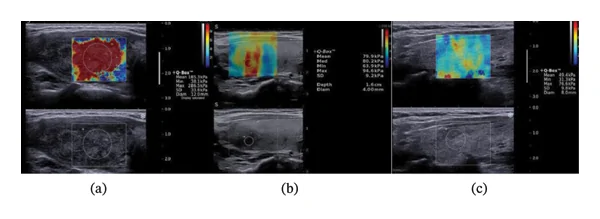
Representative sonoelastography images, taken in SAT at baseline and during the recovery phase ((a) baseline; (b) 4 weeks; (c) 8 weeks)).

SWE has been shown to be effective in monitoring SAT treatment. According to our results, thyroid stiffness decreased alongside the inflammatory syndrome (Table 3), which allows us to state that sonoelastography monitoring by measuring EI can be a useful addition in treatment adjustment (Figure 4).

Although in most cases, clinical, biochemical, and 2D ultrasonography findings are enough to provide an accurate diagnosis, in certain unclear situations, sonoelastography can be helpful in differentiating SAT [20].

The usefulness of sonoelastography in autoimmune thyroid diseases has been the subject of many studies. The first study published by Sporea et al. showed the utility of acoustic radiation force impulse to evaluate the elasticity of thyroid tissue among patients with CAT and Graves’ disease and in subjects without thyroid pathology [21]. Compared to healthy subjects, CAT has been shown to be associated with increased thyroid stiffness. Later, the same working group expanded the research by using 2D‐SWE sonoelastography [22]. They established that the best cut‐off value for predicting diffuse thyroid pathology by SWE is 22.3 kPa (AUROC = 0.71; *p* < 0.001; 59.6% Se, 76.9% Sp). In a group of patients examined by Ruchala et al. [19], the mean thyroid stiffness value assessed quantitatively as Young’s E module was 36.15 ± 18.7 kPa, while in patients studied by Margi et al. [16], the mean value was 24.0 ± 10.5 kPa. According to literature data, the 2D‐SWE appearance in CAT is different from that obtained in SAT. Ruchala, in his studies, showed that the stiffness of the thyroid tissue increases in CAT and remains unchanged despite the initiation of treatment with L‐thyroxine [18]; while in SAT, during recovery, echogenicity and vascularity usually normalize, without leaving residual changes [20]. 2D‐SWE imaging may improve monitoring of the evolution of SAT, and not least its treatment, by allowing a more accurate estimation of the optimal time for drug dose reduction [18].

SAT can sometimes mimic malignant thyroid nodules on ultrasound, leading to possible misdiagnosis. SWE helps differentiate between them by measuring tissue stiffness. Malignant nodules usually show low EI values, while SAT has much higher values. An EI cutoff of 65 kPa has been suggested to distinguish benign from malignant nodules, with 85.2% sensitivity and 93.9% specificity [23–25]. Unlike malignant nodules, which maintain stable stiffness, SAT shows a gradual decrease in stiffness as it resolves. This makes SWE useful in both diagnosis and follow‐up. Compared with thyroid nodules and CAT, where SWE has been extensively investigated, SAT remains a relatively underexplored condition in the elastography literature. The present study contributes to addressing this gap by demonstrating significantly increased thyroid stiffness during the acute phase of SAT and documenting its progressive normalization during recovery. Our findings also extend our previous work on diffuse autoimmune thyroid diseases, in which we demonstrated significantly increased thyroid stiffness in patients with CAT compared with healthy controls, with an optimal SWE cut‐off value of 22.3 kPa (AUROC = 0.71, sensitivity 59.6%, specificity 76.9%) [21, 22, 26]. In contrast to CAT, where increased stiffness generally persists despite treatment, SAT is characterized by a dynamic inflammatory process in which thyroid stiffness progressively decreases as inflammation resolves. Together, these findings support the concept that SWE is a quantitative imaging biomarker capable of reflecting disease‐specific mechanical alterations of thyroid tissue while distinguishing the distinct temporal evolution of different diffuse thyroid disorders. Additional support for the present observations comes from our previously published study on post‐COVID‐19 SAT. In that cohort, thyroid stiffness values at presentation were even higher than those observed in the present study, suggesting more severe inflammatory involvement following SARS‐CoV‐2 infection. Nevertheless, both studies demonstrated a similar temporal pattern, with progressive normalization of thyroid stiffness during corticosteroid therapy. The reproducibility of these findings across two independent SAT cohorts further supports the ability of SWE to monitor changes in thyroid tissue during recovery [27].

An important methodological strength of the present study is the excellent interobserver reproducibility obtained using a standardized SWE acquisition protocol. The ROI was systematically repositioned throughout the inflammatory lesion to identify the most representative area of consistently increased stiffness while avoiding structures known to affect elasticity measurements. Combined with minimal probe compression, standardized acquisition parameters, and independent blinded examinations performed by experienced endocrinologists, this protocol resulted in excellent interobserver agreement (ICC = 0.997; 95% CI 0.992–0.999). These findings indicate that, when a standardized protocol is applied, 2D‐SWE measurements demonstrate excellent reproducibility despite the heterogeneous distribution of inflammatory lesions in SAT.

ROC analysis demonstrated perfect discrimination between patients with SAT and healthy controls in our cohort. Although an AUC of 1.00 is biologically plausible given the marked differences in thyroid stiffness observed between active SAT and normal thyroid tissue, this result should be interpreted cautiously. The study compared patients with active SAT exclusively with healthy controls and did not include patients with other diffuse thyroid diseases that commonly enter the differential diagnosis, such as Hashimoto’s thyroiditis or Graves’ disease. Consequently, the observed diagnostic performance may partly reflect spectrum effects and should not be interpreted as evidence that SWE alone can reliably distinguish SAT from all other thyroid disorders. Nevertheless, bootstrap analysis using BCa confidence intervals confirmed the robustness of the AUC estimate within the studied cohort.

From a clinical perspective, 2D‐SWE should not be considered a replacement for conventional clinical, biochemical, and ultrasonographic evaluation. Instead, it should be regarded as a complementary quantitative imaging biomarker. Its greatest clinical value may lie in situations where the diagnosis is uncertain, such as atypical or painless presentations of SAT, equivocal conventional ultrasonographic findings, or during follow‐up, where objective quantification of thyroid stiffness may complement biochemical markers. The observation that thyroid stiffness normalized more slowly than ESR and CRP further suggests that SWE may provide additional information regarding structural tissue recovery and could potentially assist clinicians in monitoring disease evolution and treatment response.

### 4.1. Limitations and Future Research

A recognized limitation is represented by the small number of subjects and the fact that SWE offers a fairly wide range of values, where the SD is quite large. Future studies are needed to better explore these correlations and understand whether persistently increased stiffness during recovery from SAT predicts long‐term thyroid pathology, hypothyroidism, for instance. Notwithstanding the remarkable diagnostic performance noted in this study, it is imperative to emphasize that the outcomes are contingent upon a particular sample. Confirming the therapeutic usefulness of this cutoff value across various demographics and its generalizability requires external validation using bigger and more diverse cohorts. Furthermore, it is uncommon to achieve complete diagnostic accuracy, and real‐world clinical circumstances sometimes involve significant variability. Thus, the goal of future research should be to evaluate this cutoff’s resilience in multicenter, prospective trials. The histopathological correlation was not available in our cohort, and the interpretation of stiffness patterns may vary depending on lesion heterogeneity and the stage of inflammation. Previous studies have shown that SAT lesions are unevenly distributed within the gland, and this patchy distribution may influence SWE measurements [2]. Furthermore, SWE data acquisition can be affected by operator technique, probe pressure, and ROI selection.

As a next step in our investigation, we plan to compare patients with SAT to those with other diffuse thyroid pathologies, rather than healthy controls. This approach would more accurately reflect the real‐world diagnostic challenges faced by clinicians. Furthermore, a cutoff value derived from this comparison would likely be more valuable in differentiating between different thyroid conditions in practice, where subtle differences in tissue stiffness may play a crucial role in diagnosis and management. However, this does not negate the value of our current study. Establishing a clear differentiation between healthy tissue and SAT provides a strong foundation. Moving forward, focusing on diffuse thyroid pathologies could enrich the clinical impact of our future work. Controls were healthy individuals; future studies should include relevant differential diagnoses (Hashimoto’s thyroiditis, Graves’ disease).

## 5. Conclusions

SAT is associated with markedly increased thyroid stiffness that can be quantitatively assessed using 2D‐SWE. During recovery, thyroid stiffness decreases progressively and more slowly than inflammatory biomarkers, suggesting that 2D‐SWE may provide complementary information regarding structural tissue healing.

Although our findings support the potential role of 2D‐SWE as an adjunctive imaging biomarker for the longitudinal assessment of SAT, further multicenter studies including clinically relevant differential diagnoses are required before its routine clinical implementation.

## Author Contributions

Conceptualization: Ioana Golu and Mihaela Vlad; methodology: Ioana Golu and Ana Corlan; validation: Ioan Sporea and Alina Popescu; formal analysis: Ioana Golu, Ana Corlan, and Daniel Luches; investigation: Ioana Golu, Melania Balas, Dana Amzar, Natalia Milos, and Mihaela Vlad; resources: Dana Amzar, Natalia Milos, and Ioana Golu; data curation: Mervat Matei and Ioana Golu; writing–original draft preparation: Ioana Golu, Ana Corlan, and Mervat Matei; writing–review and editing: Ioana Golu, Melania Balas, and Dana Amzar: visualization Mihaela Vlad, Ioan Sporea, and Alina Popescu: supervision Mihaela Vlad and Melania Balas; project administration and funding acquisition: Ioana Golu.

## Funding

The authors acknowledge “Victor Babes” University of Medicine and Pharmacy, Timisoara, Romania, for the financial support in covering the costs of publication for this research paper.

## Disclosure

All authors have read and agreed to the published version of the manuscript.

## Ethics Statement

The study was conducted according to the guidelines of the Declaration of Helsinki and was approved by the Ethics Committee of the County Emergency Hospital Timisoara (470/20.06.2024).

## Consent

Written informed consent was obtained from all subjects involved in the study.

## Conflicts of Interest

The authors declare no conflicts of interest.

## Data Availability

The data presented in this study are available on request from the corresponding author.

## References

[1] Lanzo N. , Bohdan P. , Maria Fazzino G. F. et al., The Old and the New in Subacute Thyroiditis: An Integrative Review, Endocrines. (2022) 3, 391–410, 10.3390/endocrines3030031.

[2] Stasiak M. and Lewiński A. , New Aspects in the Pathogenesis and Management of Subacute Thyroiditis, Reviews in Endocrine & Metabolic Disorders. (2021) 22, no. 4, 1027–1039, 10.1007/s11154-021-09648-y. PMC8096888 33950404

[3] Yoo J. L. and Dong W. K. , Sonographic Characteristics and Interval Changes of Subacute Thyroiditis, Journal of Ultrasound in Medicine. (2016) 35, no. 8, 1653–1659. 10.7863/ultra.15.09049 27302899

[4] Cappelli C. , Pirola I. , Gandossi E. , Formenti A. M. , Agosti B. , and Castellano M. , Ultrasound Findings of Subacute Thyroiditis: A Single Institution Retrospective Review, Acta Radiologica. (2014) 55, no. 4, 429–433. 10.1177/0284185113498721 23969266

[5] Stasiak M. , Michalak R. , and Lewinski A. , Thyroid Primary and Metastatic Malignant Tumours of Poor Prognosis May Mimic Subacute Thyroiditis-Time to Change the Diagnostic Criteria: Case Reports and a Review of the Literature, BMC Endocrine Disorders. (2019) 19, no. 1. 10.1186/s12902-019-0415-y PMC6683567 31387553

[6] Gennisson J. L. , Deffieux T. , Fink M. , and Tanter M. , Ultrasound Elastography: Principles and Techniques, Diagnostic and Interventional Imaging. (2013) 94, no. 5, 487–495, 10.1016/j.diii.2013.01.022. 23619292

[7] Rago T. , Santini F. , Scutari M. et al., Elastography: New Developments in Ultrasound for Predicting Malignancy in Thyroid Nodules, Journal of Clinical Endocrinology and Metabolism. (2007) 92, no. 8, 2917–2922. 10.1210/jc.2007-0641 17535993

[8] Bercoff J. , Tanter M. , and Fink M. , Supersonic Shear Imaging: A New Technique for Soft Tissue Elasticity Mapping, 2004 Elasticity Mapping, IEEE Transactions on Ultrasonics, Ferroelectrics, and Frequency Control. (2004) 51, no. 4, 396–409. 10.1109/tuffc.2004.1295425 15139541

[9] Cosgrove D. , Barr R. , Bojunga J. et al., WFUMB Guidelines and Recommendations on the Clinical Use of Ultrasound Elastography: Part 4. Thyroid, Ultrasound in Medicine and Biology. (2017) 43, no. 1, 4–26, 10.1016/j.ultrasmedbio.2016.06.022. 27570210

[10] Bamber J. , Cosgrove D. , Dietrich C. F. et al., EFSUMB Guidelines and Recommendations on the Clinical Use of Ultrasound Elastography. Part 1: Basic Principles and Technology, Ultraschall in der Medizin. (2013) 34, no. 2, 169–184, 10.1055/s-0033-1335205. 23558397

[11] Cantisani V. , Lodise P. , Grazhdani H. et al., Ultrasound Elastography in the Evaluation of Thyroid Pathology. Status, European Journal of Radiology. (2014) 83, no. 3, 420–428, 10.1016/j.ejrad.2013.05.008. 23763859

[12] Sebag F. , Vaillant-Lombard J. , Berbis J. et al., Shear Wave Elastography: A New Ultrasound Imaging Mode for the Differential Diagnosis of Benign and Malignant Thyroid Nodules, Journal of Clinical Endocrinology and Metabolism. (2010) 95, no. 12, 5281–5288, 10.1210/jc.2010-0766. 20881263

[13] Liu J. , Zhang Y. , Ji Y. , Wan Q. , and Dun G. , The Value of Shear Wave Elastography in Diffuse Thyroid Disease, Clinical Imaging. (2018) 49, 187–192, 10.1016/j.clinimag.2018.03.019. 29627743

[14] Tranquart F. , Bleuzen A. , Pierre-Renoult P. et al., Elastosonography of Thyroid Lesions, Journal de Radiologie. (2008) 89, no. 1 Pt 1, 35–39. 10.1016/s0221-0363(08)70367-6 18288024

[15] Rubaltelli L. , Corradin S. , Dorigo A. et al., Differential Diagnosis of Benign and Malignant Thyroid Nodules at Elastosonography, Ultraschall in der Medizin. (2009) 30, no. 2, 175–179. 10.1055/s-2008-1027442 18496776

[16] Magri F. , Chytiris S. , Capelli V. et al., Shear Wave Elastography in the Diagnosis of Thyroid Nodules: Feasibility in the Case of Coexistent Chronic Autoimmune Hashimoto’s Thyroiditis, Clinical Endocrinology. (2012) 76, no. 1, 137–141, 10.1111/j.1365-2265.2011.04170.x. 21740455

[17] Bhatia K. S. , Tong C. S. , Cho C. C. M. , Yuen E. H. Y. , Lee Y. Y. P. , and Ahuja A. T. , Shear Wave Elastography of Thyroid Nodules in Routine Clinical Practice: Preliminary Observations and Utility for Detecting Malignancy, European Radiology. (2012) 22, no. 11, 2397–2406, 10.1007/s00330-012-2495-1. 22645042

[18] Ruchala M. , Szczepanek E. , and Sowinski J. , Sonoelastography in de Quervain Thyroiditis, Journal of Clinical Endocrinology and Metabolism. (2011) 96, no. 2, 289–290, 10.1210/jc.2010-1595. 21296992

[19] Ruchala M. , Szczepanek-Parulska E. , Zybek A. et al., The Role of Sonoelastography in Acute, Subacute, and Chronic Thyroiditis: A Novel Application of the Method, European Journal of Endocrinology. (2012) 166, no. 3, 425–432, 10.1530/eje-11-0736. 22143319

[20] Park S. Y. , Kim E. K. , Kim M. J. et al., Ultrasonographic Characteristics of Subacute Granulomatous Thyroiditis, Korean Journal of Radiology. (2006) 7, no. 4, 229–234, 10.3348/kjr.2006.7.4.229. PMC2667608 17143025

[21] Sporea I. , Vlad M. , Bota S. et al., Thyroid Stiffness Assessment by Acoustic Radiation Force Impulse Elastography (ARFI), Ultraschall in der Medizin. (2011) 32, no. 3, 281–285, 10.1055/s-0029-1246048. 21321841

[22] Vlad M. , Golu I. , Bota S. et al., Real-Time Shear Wave Elastography May Predict Autoimmune Thyroid Disease, Wiener Klinische Wochenschrift. (2015) 127, no. 9-10, 330–336, 10.1007/s00508-015-0754-2. 25835593

[23] Bhatia K. S. , Tong C. S. , Cho C. C. , Yuen E. H. Y. , Lee J. , and Ahuja A. T. , Reliability of Shear Wave Ultrasound Elastography for Neck Lesions Identified in Routine Clinical Practice, Ultraschall in der Medizin. (2012) 33, no. 5, 463–468, 10.1055/s-0032-1325330. 23070932

[24] Lim D. J. , Luo S. , Kim M. H. , Ko S. H. , and Kim Y. , Interobserver Agreement and Intraobserver Reproducibility in Thyroid Ultrasound Elastography, American Journal of Roentgenology. (2012) 198, no. 4, 896–901, 10.2214/ajr.11.7009. 22451558

[25] Hu X. , Liu Y. , and Qian L. , Diagnostic Potential of real-time Elastography (RTE) and Shear Wave Elastography (SWE) to Differentiate Benign and Malignant Thyroid Nodules: A Systematic Review and Meta-Analysis, Medicine (Baltimore). (2017) 96, no. 43, 10.1097/md.0000000000008282. PMC5671829 29068996

[26] Matei M. , Vlad M. M. , Golu I. , Dumitru C. Ș. , De Scisciolo G. , and Matei S. C. , Can Routine Laboratory Tests Be Suggestive in Determining Suspicions of Malignancy in the Case of Thyroid Nodules?, Medicina. (2023) 59, no. 8, 10.3390/medicina59081488. PMC10456539 37629778

[27] Golu I. , Balas M. , Amzar D. , Milos I. , Corlan A. , and Vlad M. , The Value of Shear-Wave Elastography in the Diagnosis and Follow-Up of Post-COVID-19 Subacute Thyroiditis, Ultrasound in Medicine and Biology. (2022) 48, 10.1016/j.ultrasmedbio.2022.04.128.

